# Use and Misuse of Cost-Effectiveness Analysis Thresholds in Low- and Middle-Income Countries: Trends in Cost-per-DALY Studies

**DOI:** 10.1016/j.jval.2017.12.016

**Published:** 2018-07

**Authors:** Ashley A. Leech, David D. Kim, Joshua T. Cohen, Peter J. Neumann

**Affiliations:** Center for the Evaluation of Value and Risk in Health, Institute for Clinical Research and Health Policy Studies, Tufts Medical Center, Boston, MA, USA

**Keywords:** cost effectiveness, DALY, LMICs, thresholds, willingness to pay

## Abstract

**Objectives:**

To determine what thresholds are most often cited in the cost-effectiveness literature for low- and middle-income countries (LMICs), given various recommendations proposed and used in the literature to date, and thereafter to assess whether studies appropriately justified their use of threshold values.

**Methods:**

We reviewed the contents of the Tufts Medical Center Global Health Cost-Effectiveness Analysis Registry, a repository of all English language cost-per-disability-adjusted life-year averted studies indexed in PubMed. Our review included all catalogued cost-per-disability-adjusted life-year studies published from 2000 through 2015. We restricted attention to studies that investigated interventions in LMICs.

**Results:**

Our analysis identified 381 studies (80%) focused on LMICs. Of these studies, 250 (66%) cited the World Health Organization’s 1 to 3 times gross domestic product per capita threshold. A full-text review of 60 (24%) of these articles (randomly selected) revealed that none justified use of this threshold in the particular country or countries studied beyond citing (generic) guideline documents.

**Conclusions:**

Cost-effectiveness analysis can help inform health care spending, but its value depends on incorporating assumptions that are valid for the applicable setting. Rather than rely on commonly used, generic economic thresholds, we encourage authors to use context-specific thresholds that reflect local preferences.

## Why Cost-Effectiveness Thresholds Are Important

Nearly 40 years ago, the World Health Organization (WHO) promoted the use of cost-effectiveness analysis (CEA) to evaluate health intervention programs for low- and middle-income countries (LMICs) [1]. CEA compares an intervention’s costs and benefits in terms of the incremental cost-effectiveness ratio (ICER), which is calculated as its incremental cost (compared with some alternative, such as the previous standard of care) divided by its incremental effectiveness (e.g., price per unit benefit). Health effects in global health are typically measured in terms of disability-adjusted life-years (DALYs) averted, a metric that originated to quantify the global burden of disease [2], [3], [4]. A DALY reflects both changes in life expectancy and quality of life (pain, function, or both).

Because an ICER can be thought of as the “price” at which an intervention produces health gains, that price must be compared with a benchmark (or “threshold,” e.g., $10,000/DALY averted) to determine whether the intervention is “cost-effective.” If the ICER falls below the threshold, the intervention is said to be favorably cost-effective, because it averts each DALY at a low cost. ICERs exceeding the threshold are considered unfavorable. Because the threshold represents what society is willing to pay for health gains and, correspondingly, what goods and services it would be willing to forego for these gains, its assumed value is important. If total health care spending is fixed, implying that other health care interventions must be eliminated to pay for the adoption of a new intervention, the new intervention’s ICER should be at least as favorable as the ICER for interventions that are cut. Even if health care spending can grow, increased health care spending necessitates cuts to other categories of consumption. In this circumstance, the threshold represents the value of consumption outside the health care sector that individuals are willing to give up to avert one more DALY.

## Evolution of Cost-Effectiveness Thresholds for LMICs

The World Bank first introduced a set of thresholds in 1993 by classifying ICERs as “highly cost-effective” if they fell below $50/DALY averted in low-income settings or below $150/DALY averted in middle-income settings; it classified ICERs ranging from $150 to $200/DALY averted as cost-effective [5]. The WHO's Commission on Macroeconomics and Health (CMH) report first used 'per capita income' to estimate the economic loss resulting from the burden of diseases across countries. This report, however, did not intend to endorse a cost-effectiveness threshold. However, the WHO's Choosing Interventions that are Cost-Effective (CHOICE) program later adopted the CMH report values to define interventions whose ICERs fell between 1 to 3 times a country's annual gross domestic product (GDP) per capita per DALY averted as “cost-effective” [2], [6], [7], [8], [9].

With various recommendations having been proposed over the years, we set out to determine what thresholds are most often used in the cost-effectiveness literature for LMICs and whether the thresholds cited have changed over time. After having identified the most commonly used threshold, we reviewed a subset of CEAs to assess whether the studies justified their use of that particular benchmark value.

## Global Health Cost-Effectiveness Analysis Registry

We reviewed the contents of the Tufts Medical Center Global Health Cost-Effectiveness Analysis (GHCEA) Registry, a repository of all English language cost-per-DALY averted studies indexed in PubMed [10]. The GHCEA Registry is a publicly available online resource sponsored by the Bill and Melinda Gates Foundation (www.ghcearegistry.org). We included all catalogued studies published from 2000 through 2015. We restricted attention to studies that investigated interventions in LMICs.

## Classification of Cost-Effectiveness Thresholds

To identify the threshold values LMIC cost-effectiveness articles use most often and determine whether usage has changed over time, we first assigned each threshold value to one of five categories: 1) 1 to 3 times GDP per capita, cited in the past by the WHO in its World Health Report and in its guidelines on Choosing Interventions that are Cost-Effective [6], [9]; 2) “highly cost-effective” and “cost-effective,” as defined by the World Bank’s 1993 World Development Report for low- and middle-income settings; 3) country-specific thresholds; 4) no threshold mentioned; and 5) other values, including studies citing multiple threshold categories. We then examined changes in the citation frequency of these thresholds over time. Finally, we randomly selected 60 (24%) studies that cited the most commonly referenced threshold value (which turned out to be 1–3 times GDP per capita, as described later) and reviewed the full text of these articles to determine how often they provided a rationale for this threshold value.

## Trends in the Use of Cost-Effectiveness Thresholds, 2000 to 2015

We identified 479 cost-per-DALY averted studies that collectively reported 3859 cost-per-DALY ratios from the GHCEA Registry. Details on these studies are provided elsewhere [10]. Of the 479 cost-per-DALY averted studies, 381 (80%) focused on LMICs. Studies that reported on LMIC interventions were most likely to cite 1 to 3 times GDP per capita as the cost-effectiveness threshold (*P* < 0.0001). A total of 66% (N = 250) specified only this threshold, whereas 23% of studies did not specify any threshold value (N = 89) (Table 1).

**Table 1:** Characteristics of published cost-per-DALY studies for LMICs, 2000–2015

**By threshold**	**2000–2015 analyses (N = 381) (%)**
1–3 times country’s GDP only	250 (66%)
WB/WHO 1990s only	15 (4%)
Country-specific only	0 (0%)
None	89 (23%)
Other*	27 (7%)
By region	
Latin America and Caribbean	44 (12%)
Sub-Saharan Africa	156 (41%)
Southeast Asia, East Asia, and Oceania	58 (15%)
South Asia	42 (11%)
North Africa and Middle East	10 (3%)
Central Europe, Eastern Europe, and Central Asia	9 (2%)
Other†	62 (16%)
By author country affiliation‡	Missing = 6
High income	294 (78%)
1–3 times country’s GDP only	187 (64%)
WB/WHO 1990s only	14 (5%)
Country-specific only	0 (0%)
None	70 (24%)
Other*	23 (8%)
LMICs	81 (22%)
1–3 times country’s GDP only	58 (72%)
WB/WHO 1990s only	1 (1%)
Country-specific only	0 (0%)
None	19 (23%)
Other*	3 (4%)

DALY, disability-adjusted life-year; GDP, gross domestic product; LMIC, low- and middle-income country; WB, World Bank; WHO, World Health Organization.

⁎ Includes studies citing multiple threshold categories and other values not included in the other categories.

† Includes studies that did not specify any threshold value.

‡ We used primary author’s affiliated university or membership during time of publication as a proxy for author country affiliation.

**Fig. 1:**
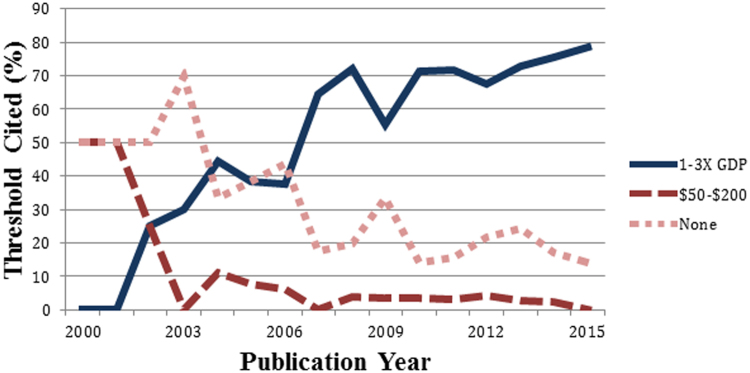
Thresholds cited in LMIC cost/DALY averted studies (% by year). DALY, disability-adjusted life-year; GDP, gross domestic product; LMIC, low- and middle-income country.

Since 2000, the number of LMIC cost-per-DALY studies published each year has increased substantially, from 4 studies in 2000 to 70 published in 2015. The proportion of LMIC studies citing a threshold of 1 to 3 times GDP per capita increased over time, 10% of all studies in 2000 to 2002 to 76% in 2013 to 2015, whereas the proportion citing the 1993 World Development Report threshold of $50 to $200 or mentioning no threshold declined (Fig. 1). Before the publication of WHO’s World Health Report and its guidelines on Choosing Interventions that are Cost-Effective in the early 2000s, both of which recommended a threshold value of 1 to 3 times GDP per capita, many studies cited no threshold or mentioned other cost-effectiveness thresholds. Finally, all LMIC studies citing the 1 to 3 times GDP per capita threshold for which we conducted a full-text review referenced only the CMH report, the WHO guideline, or other literature as justification; none provided an explicit rationale for that threshold value.

## Summary and Policy Implications

The growing use of the 1 to 3 times GDP per capita threshold raises concerns because it lacks a foundation [2], [7], [8], [11]. Although this threshold range roughly corresponds to what has become convention for high-income countries, such as the United States [12], per capita consumption in wealthier countries exceeds LMIC per capita consumption by 1 to 2 orders of magnitude; hence, extrapolation to LMICs is substantial and thus may not be valid. Indeed, analysts have argued that the threshold should be lower for both low-/middle-income (e.g., 0.01–0.51 times GDP per capita) and middle-/high-income (e.g., 0.18–0.71 times GDP per capita) [11] countries.

Given that the WHO provided no clear rationale for the 1 to 3 times GDP per capita cost-effectiveness threshold, it is perhaps unsurprising that none of the CEAs we reviewed did so either. Instead of relying on the 1 to 3 times GDP per capita as a convention, the global health economics field should develop context-specific thresholds corresponding to opportunity cost. In line with recent analyses, the routinely used threshold of 1 to 3 times GDP per capita is too high and is more salient for LMICs that have more stringent resource constraints [11], [13]. Because of differences in culture, resource constraints, and data availability, threshold and valuation estimates should not be equivalent across economies [2].

If new health care spending tends to displace other health care spending—as is often the case in countries with a single (often public) payer with a fixed budget, the opportunity cost is the health loss resulting from cuts to displaced health care. New spending is worthwhile if it averts more DALYs than the old spending; equivalently, the new health care intervention’s ICER must be more favorable than the ICER for displaced health care. Assuming that new health care spending displaces the least efficient currently funded intervention in a particular country, the ICER for the new health care must be at least as favorable as the *least* favorable ICER among currently funded interventions. The least efficient intervention’s ICER—that is, the ICER “at the margin”—hence represents the ICER threshold for new health care spending in that country. If, alternatively, health care spending is not fixed (as is more typical in settings with decentralized health care), the opportunity cost is the eliminated non–health care consumption. In these cases, shifting spending to health care is worthwhile if the averted DALYs are worth more than the lost consumption; that is, the appropriate threshold corresponds to the societal willingness to pay to avert one DALY.

Ideally and theoretically, each country should develop its own cost-effectiveness thresholds to inform resource allocation and preferences reflecting the health care system at large. Although we acknowledge the practical limitations of this task, our findings represent a call to action for key international agencies to re-examine standard criteria for designating an intervention as having reasonable value for investment.

## Conclusions

The range of opinions on threshold values has motivated calls for developing a consensus [2], [7], [11], [13], [14], [15], [16]. CEA can help inform health care spending, but its value depends on using assumptions that are appropriate to the analysis setting. Extrapolation of the commonly used threshold of 1 to 3 times GDP per capita to LMICs is problematic given their pronounced resource constraints and contextual considerations that must be considered when placing value on health. The benchmark defining acceptable value is a key assumption. Rather than relying on generic global benchmarks, we encourage practitioners to develop context-specific values reflecting the health care system and local priorities.

Source of financial support: This study was funded by the Bill and Melinda Gates Foundation (grant no. OPP1097194).
